# Effect of *Arthrospira (Spirulina) maxima* Supplementation and a Systematic Physical Exercise Program on the Body Composition and Cardiorespiratory Fitness of Overweight or Obese Subjects: A Double-Blind, Randomized, and Crossover Controlled Trial

**DOI:** 10.3390/md16100364

**Published:** 2018-10-01

**Authors:** Marco Antonio Hernández-Lepe, José Alberto López-Díaz, Marco Antonio Juárez-Oropeza, Rosa Patricia Hernández-Torres, Abraham Wall-Medrano, Arnulfo Ramos-Jiménez

**Affiliations:** 1 Instituto de Ciencias Biomédicas, Universidad Autónoma de Ciudad Juárez, Ciudad Juárez 32310, Chihuahua, Mexico; marco.hernandez@uacj.mx (M.A.H.-L.); joslopez@uacj.mx (J.A.L.-D.); awall@uacj.mx (A.W.-M.); 2 Departamento de Bioquímica, Facultad de Medicina, Universidad Nacional Autónoma de Mexico, Ciudad de Mexico 04510, Mexico; majo_ya@yahoo.com.mx; 3 Facultad de Ciencias de la Cultura Física, Universidad Autónoma de Chihuahua, Ciudad Juárez 32310, Chihuahua, Mexico; rphernant@yahoo.com

**Keywords:** Overweight, obesity, body fat, maximal oxygen uptake, double-blind, randomized controlled trial

## Abstract

Excess weight and obesity are major risk factors for many chronic diseases, and weight-loss interventions often include systematic exercise and nutritional supplements. The purpose of this study was to determine the independent/synergistic effects of *Arthrospira (Spirulina) maxima* supplementation (six weeks, 4.5 g·day^−1^) and a systematic physical exercise program (six weeks, twice weekly) on the body composition and cardiorespiratory fitness of overweight and obese subjects. To achieve this, 27 overweight and 25 obese sedentary male subjects were assigned to four interventions through a randomized double-blind, crossover controlled trial: A physical exercise program, with (SE) or without (Ex) *Spirulina maxima*; or no-exercise program, with (Sm) and without (C) *Spirulina maxima*. The body composition and cardiorespiratory fitness parameters were taken during a maximum intensity test. As compared to the C group, the body fat percentage of the SE, Sm and Ex groups was reduced (*p* < 0.05), while their maximal oxygen uptake improved (*r* = −0.40), and obese subjects benefited more significantly. Weight loss, the time to reach fatigue and the onset of blood lactate accumulation were improved in both of the *Spirulina maxima* supplemented groups, regardless of the subjects’ body weight. *Spirulina maxima* supplementation synergistically improves the effects of systematic exercise on body composition and cardiorespiratory fitness parameters in overweight, but mostly in individuals with obesity. Trial registration: Clinical Trials, NCT02837666. Registered 19 July 2016.

## 1. Introduction

Excess weight and obesity (body mass index (BMI) ≥ 25 kg·m^−2^) are major risk factors for many non-communicable chronic diseases, including type 2 diabetes, cardiovascular diseases (CVD), and cancer [1]. Behavioral-based interventions, focused on promoting a healthy diet and physical activity, have proven to be effective in reducing people’s body weight (BW) and body fat stores [2]. Systematic exercise improves people’s general fitness—e.g., higher maximal oxygen uptake (VO_2_max), a prolonged onset of blood lactate accumulation (OBLA), a better ventilatory anaerobic threshold, and an overall improvement of resting heart rate (RHR) and body fat mobilization [3,4].

However, behavioral-based interventions are even more effective when combined with prescribed drugs or nutritional supplements, aimed to reduce the body’s fat stores and energy expenditure [5,6]. Marine product-based supplements are important sources of bioactive compounds [7]. Spirulina is a group of cyanobacteria that is cultivated around the world and belongs to the *Spirulina* and *Arthrospira* genera, consisting of about 15 species; *Arthrospira maxima* is the most common specie, and most of the published research on worldwide public health refers to this specific specie, although they have been published under the name of “*Spirulina*” [8]. The cyanobacteria used in this research belongs to the specie, *Arthrospira maxima*, but for the purpose of the clinical trial, we refer collectively to both as ‘*Spirulina maxima* (*S. maxima*)’.

*Spirulina maxima* is a marine nutritional/nutraceutical supplement due to its content of phytochemicals (phenolic compounds, carotenoids, and tocopherols) and essential nutrients (proteins, n-3 and n-6 fatty acids) [9]. Recently, studies reporting on the use of *S. maxima* have been demonstrating the biological potential of its compounds, including, but not restricted to, its antioxidant and hypolipidemic activity. This is due to the interaction of its phytochemicals with free radicals that inhibit lipid peroxidation, an action related to prevention of CVD, some types of cancer, inflammatory diseases, diabetes mellitus, and non-alcoholic fatty liver disease [10,11]. However, most studies have been conducted in animal models, with scarce studies focused on its effects on humans. Seo et al. [12] have shown smaller adipose depots, lower blood lipid concentrations, and lower body mass gain in mice administered with a high-fat diet (HFD) and *S. maxima* than in an HFD mice control. Szulinska et al. [13] found a decrease in body mass, waist circumference, plasma lipid levels, inflammation, and oxidative stress in hypertensive obese patients with *S. maxima* supplementation (2 g for three months). However, controlled studies focused on the effect of the administration of *S. maxima* and a systematic exercise program in humans, have not yet been reported [14].

Therefore, the goal of this study was to evaluate the independent and synergistic effect of *S. maxima* supplementation, with or without a systematic physical exercise program, on body composition (BW and body fat percentage (%BF)) and cardiorespiratory fitness (VO_2_max, anaerobic threshold, RHR, OBLA, and time to fatigue (TF)) parameters. The differential effects in overweight and obese male adults are also reported. We hypothesize that *S. maxima* intake and a systematic physical exercise program decrease BW and %BF, and improve VO_2_max, RHR, OBLA, and TF, in overweight and male patients with obesity.

## 2. Results

**Figure 1:**
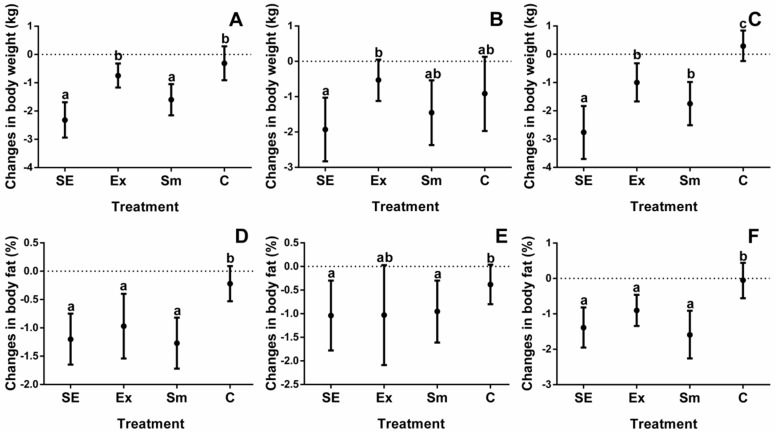
Changes in body weight and body fat percentage by treatments. SE: *Spirulina* and exercise; Ex: exercise and placebo; Sm: *Spirulina* without exercise; and C: control (placebo treatment). (**A**) Total body weight changes of subjects; (**B**) Body weight changes in overweight subjects; (**C**) Body weight changes in subjects with obesity; (**D**) Total body fat changes of subjects; (**E**) Body fat changes in overweight subjects; and (**F**) Body fat changes in subjects with obesity. Data are shown as the mean, with 95% confidence intervals. Different letters indicate the statistical differences between groups (*p* < 0.05).

**Figure 2:**
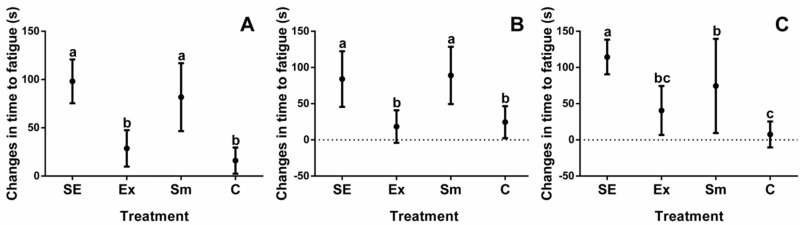
Changes in time to fatigue by treatments. SE: *Spirulina* and exercise; Ex: exercise and placebo; Sm: *Spirulina* without exercise; and C: control (placebo treatment). (**A**) Total time to fatigue changes of subjects; (**B**) Time to fatigue changes in overweight subjects; and (**C**) Time to fatigue changes in obesity subjects. Data are shown as the mean, with 95% confidence intervals. Different letters indicate the statistical differences between groups (*p* < 0.05).

**Figure 3:**
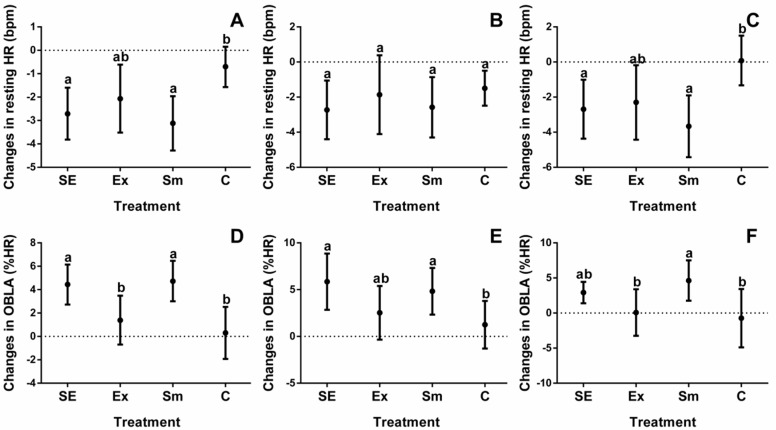
Changes in resting heart rate and onset blood lactate accumulation by treatments. HR: heart rate; OBLA: onset blood lactate accumulation; %HR: percentage of maximal heart rate; SE: *Spirulina* and exercise; Ex: exercise and placebo; Sm: *Spirulina* without exercise; and C: control (placebo treatment). (**A**) Total resting HR changes of subjects; (**B**) Resting HR changes in overweight subjects; (**C**) Resting HR changes in obesity subjects; (**D**) Total OBLA changes of subjects; (**E**) OBLA changes in overweight subjects; and (**F**) OBLA changes in obesity subjects. Data are presented as the mean with 95% confidence intervals. Different letters indicate the statistical differences between groups (*p* < 0.05).

**Figure 4:**
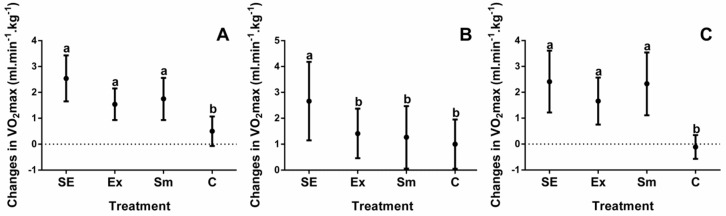
Changes in maximal oxygen uptake by treatments. VO_2_max: Maximal oxygen uptake; SE: *Spirulina* and exercise; Ex: exercise and placebo; Sm: *Spirulina* without exercise; and C: control (placebo treatment). (**A**) Total VO_2_max changes of subjects; (**B**) VO_2_max changes in overweight subjects; and (**C**) VO_2_max changes in subjects with obesity. Data are shown as the mean, with 95% confidence intervals. Different letters indicate the statistical differences between groups (*p* < 0.05).

Baseline measurements of study participants are shown in Table 1, while results relating to body composition and cardiorespiratory fitness of all four intervention groups are depicted in Figure 1, Figure 2, Figure 3 and Figure 4.

Individual differences are often wide ranging, for that reason the results are shown as differences between the initial evaluations and after treatments. Additionally, since one individual (either performing or not in the exercise protocol) underwent a crossover trial, with or without *S. maxima*, all changes, depicted in Figure 1, Figure 2, Figure 3 and Figure 4, were corrected for the basal BW and BMI.

### 2.1. Diet

The daily energy intake during the six weeks of supplementation showed no statistically significant differences (*p* < 0.05) between the treatments for dietary variables at the beginning (2054 ± 104 kcal·day^−1^) compared with those at the end of the study (2146 ± 98 kcal·day^−1^). No adverse effects of dietary or *S. maxima* supplementation were reported during the study.

### 2.2. Body Composition

Initially, individuals with obesity had a higher BMI (5.9 kg·m^−2^) and %BF (8.4%) than overweight individuals (Table 1). According to Figure 1A, BW changes were more evident (*p* < 0.05) in *S. maxima*-supplemented groups (−2.2 kg (SE) and −1.6 kg (Sm)) than those observed for non-supplemented ones (−0.8 kg (Ex) and −0.3 kg (C)), indicating that subjects with obesity (Figure 1C) were more benefited than those with overweight (Figure 1B). The %BF of the SE, Sm, and Ex groups was also reduced (*p* < 0.05) (Figure 1D), regardless of the subjects’ BW status. Lastly, according to Table 2, body weight change (ΔBW) was statistically (*p* < 0.05) related to changes in body fat (*r* = 0.3) and RHR (*r* = 0.3), and to TF (*r* = 0.5) and VO_2_max (*r* = −0.5) during the maximal intensity test (MIT).

### 2.3. Cardiorespiratory Fitness

The maximal oxygen uptake (VO_2_max) in overweight subjects was 8.8 mL·kg^−1^·min^−1^ greater than that of individuals with obesity, but the RHR in both groups was not different (Table 1). Changes in TF by treatments (Figure 2) mainly favored subjects with obesity (Figure 2C), and neither RHR (Figure 3A) nor OBLA (Figure 3D) had an apparent effect on BMI status, which was mostly improved in *S. maxima* supplemented groups (SE and Sm).

Moreover, VO_2_max improved in the SE, Sm, and Ex groups as compared to the C group (Figure 4A), and individuals with obesity (Figure 4C) were more significantly benefited than overweight ones (Figure 4B). According to Table 2, besides correlating with Δ%BW, ΔVO_2_max also correlated (*r*) with Δ%BF (−0.4), ΔTF (0.4), and ΔRHR (−0.2).

## 3. Discussion

This study is the first to focus on examining the effects of *S. maxima* supplementation in body composition in humans using a double-blind, randomized, crossover trial design. The main finding of this study on the effects of *S. maxima* supplementation (4.5 g), alone or together with systematic exercise, on body composition was a significant decrease in BW and %BF during six weeks of treatment. Concerning the cardiorespiratory parameters, it was found that there is a significant improvement in RHR, TF, VO_2_max, and %HR at OBLA with *S. maxima* supplementation and/or systematic exercise.

There exist about 15 *Spirulina* species, but only three—*Spirulina platensis* (*Arthrospira platensis*), *Spirulina fusiformis* (*Arthrospira fusiformis*), and *S. maxima*—have been intensively investigated, reporting on the similarity between their nutritional composition and potential biological effects [15]. Cultivation affects the nutritional properties of the harvested product, and the nutritional content of the raw product differs from that mentioned in the commercial labels, although in this study we carried out a proximate analysis of the product, ensuring the nutritional content (Supplementary File 1).

### 3.1. Body Composition

Studies on the potential effects of nutraceutical supplements are increasing in number. Miczke et al. [16], reported the effect of 2 g·day^−1^ of *S. maxima* supplementation on 40 hypertension subjects in a double-blind, placebo-controlled randomized trial for three months. After the intervention, they reported a significant reduction of BW, BMI, and blood pressure in the group supplemented with *S. maxima*, compared with that of the placebo group. Mazokopakis et al. [17] studied the effect of *Spirulina* supplementation (1 g·day^−1^) during three months on the BW, BMI, and lipid profile in patients with dyslipidemia and reported a significant decrease in total cholesterol, triacylglycerols, and cholesterol associated with low-density lipoproteins (LDL-C). In our study, we found a significant decrease in BW and %BF with SE and Sm in almost all treatments, compared with the control. In addition, there was a tendency of presenting an additive effect of *S. maxima* and exercise in BW. The inconsistent results in the Ex group was probably due to the existence of a considerable interindividual variability in the magnitude of weight loss produced by exercise, and a specific kind of exercise program for overweight and obese persons does not exist [18]. The *S. maxima* groups showed better results in relation to body composition, probably due to their specific action mechanism in the organism. Fujimoto et al. [19] hypothesized that some *Spirulina* compounds can reduce the macrophages infiltration into visceral fat and prevent the accumulation of liver lipids, resulting in weight reduction, specifically body fat, which would explain our clinical trial results. However, studies focused on the elucidation of the specific compounds and action mechanisms by which *Spirulina* affects body composition are necessary.

### 3.2. Cardiorespiratory Fitness

#### 3.2.1. Time to Fatigue

The effects of *S. maxima*, observed in the present study, strengthen the data reported by Kalafati et al. [20], who studied the effect of *S. maxima* supplementation (6 g·day^−1^) in nine recreational runners, training at least two days a week, 45 min per session. The runners had a significant improvement in TF in a submaximal exercise test (~40 s) after four weeks of the clinical trial. In addition to the VO_2_max increase observed here, it is probable that some of the compounds, present in *S. maxima*—such as phycocyanins, essential fatty acids, carotenoids or phenolic compounds—act individually in different metabolic processes to improve cardiorespiratory fitness. These effects are discussed in the next sections.

#### 3.2.2. Resting Heart Rate and OBLA

A high RHR has been associated with cardiovascular mortality in numerous epidemiologic studies [21,22]. Torres-Duran et al. [23] studied the effect of *S. maxima* supplementation (5 g·day^−1^) for 15 days in the lactate concentration of recreational runners (*n* = 41), before and after intensive exercise. Their results showed a trend of lowering the lactate concentration (mM) before exercise with *S. maxima* supplementation (1.7 ± 0.6), compared with exercise without supplementation (2.3 ± 2.2), and these results were similar to the lactate concentrations after exercise with *S. maxima* supplementation (12.8 ± 6.3), compared with exercise without supplementation (15.5 ± 4.2). Moreover, Torres-Duran et al. did not quantify the lactate concentrations through the exercise test, because it was not possible to determine the OBLA, but our results, showing the decrease of RHR, can be directly related only with an increase of the VO_2_max in each MIT. Additionally, even though the trial duration was short-term, we found minimum but statistical differences after treatments. Lu et al. [24] examined the effect of *Spirulina* supplementation (7.5 g·day^−1^) in lactate concentrations before maximal athletic activity in a randomized double-blind placebo-controlled study of 16 young students. They reported a statistical decrease (*p* < 0.05) in blood lactate concentration (mM) after supplementation (three weeks), compared with the control, before performing a maximal athletic activity (2.3 ± 0.3 vs. 3 ± 0.4). This study strengthens our results that a high protein content nutraceutical like *Spirulina* can improve some biochemical parameters, such as RHR and blood lactate concentration, which can be the starting point for studies focused on these response variables in different kinds of populations. Our results relating to RHR and %HR at OBLA may be due to *S. maxima* proteins exerting an anti-inflammatory role or indirectly modulating the inflammatory state and the balance of muscle cells in order to favor the biological response and the adaptation of the muscle tissue during the MIT. The possible action mechanism may involve the suppression of skeletal muscle proteolysis and the stimulation of protein synthesis due to the high quality protein content in *S. maxima* [25]. Regarding OBLA, the conclusions of different researches suggest the utilization of anaerobic exercise sessions, with high-intensity interval training (HIIT), to induce blood lactate accumulation, providing a stimulus to improve aerobic performance markers, such as OBLA [26]. Our OBLA results did not correlate with any of the other analyzed cardiorespiratory parameters due to the fact that the physiological adaptations of sedentary overweight and obese subjects to exercise may be lower than that in the active population [27].

#### 3.2.3. Maximal Oxygen Uptake

In our study, there was a low but statistical increase in VO_2_max in the SE and Sm groups, which may not be of clinical significance, since it is due to body mass loss rather than to an improvement in cardiorespiratory fitness. Similar to our results, De Strijcker et al. [28] reported on an intervention of 10 weeks of HIIT (twice a week) in 16 male overweight/obese subjects. They showed a low but statistical increase in VO_2_max (~0.2 mL·min^−1^·kg^−1^) and a decrease in BW (~1 kg). According to this study, a growing body of evidence suggests that regular exercise training improves health benefits, such as VO_2_max and body mass, but the optimal intensity and volume necessary to obtain maximal benefits remains to be defined [29]. The VO_2_ improvements can be affected by both aerobic capacity and the intensity of exercise [30], and even in our study, the training intensity of sedentary subjects accorded with that recommended by the American College of Sport Medicine (ACSM) for the improvement of cardiorespiratory fitness (>40% of VO_2_max) [27]. The ideal length and duration of studies of sedentary adults are not well defined, showing clinical effects from 20 min·day^−1^, for five weeks at 55% of VO_2_max, to 36 min·day^−1^ for 24 weeks at 50% of VO_2_max [31]. For that reason, we considered that using a training program at the moderate to high intensity of 30 min·day^−1^ for 12 weeks was appropriate for the improvement of the cardiorespiratory parameters of our study population.

The correlation between body composition and VO_2_max could be due to the exercise program effect and cyanobacteria in the following way: (1) *Spirulina* regulates lipolysis due to the adipose tissue effect, probably as a consequence of the action of its fatty acids composition [32]; and (2) the protein content in *S. maxima* (~65%) inhibits the gut lipid absorption [33], resulting in mild steatorrhea [34]. However, the research has focused mainly on animal models, so further studies are required to prove that this is similar in humans.

Our positive results regarding the cardiorespiratory parameters of the *S. maxima* intake groups suggest that the high bioavailability of antioxidant compounds, present in the cyanobacteria, could have a synergistic effect against oxidative stress caused by the MIT. Carotenoids, present in *S. maxima*, could be exceptional quenchers of reactive oxygen species, which are induced by extenuate physical activity, inhibiting lipid peroxidation [35] and resulting in an improvement of cardiorespiratory performance, manifested in a major utilization of lipids, such as energy substrates. This indicates that focusing clinical studies on the intake of *S. maxima* could result in positive effects, not only in body composition and cardiorespiratory fitness, but also in diseases related to oxidative stress.

### 3.3. Limitations

Even though the present study shows evidence of the clinical importance of *S. maxima* supplementation, there is a limitation of determining the correct action mechanisms due to the variety of compounds present in *S. maxima* [36]. On the other hand, the magnitude of the effects of nutritional treatments and physical exercise depends on various factors, including the type and quantity of the studied population, the duration of treatment, and the type and quantity of the product administered. Regarding gender, in most clinical studies women are underrepresented [37]. In this study, we included them, although only seven women finished the protocol, so they were not included in the analyses. On the other hand, with respect to the inclusion criteria, only sedentary people with obesity were selected, so the treatment may not have the same impact in other populations. Regarding the number of participants, the duration of the treatment and the quantity of *S. maxima* administered, double-blind studies have reported on the benefits of 4 weeks of treatment of 9 participants, and other studies have reported on the benefits of treatment with 1 g·day^−1^ of *S. maxima* for 12 weeks [14].

## 4. Materials and Methods

*Spirulina maxima* was obtained commercially from Alimentos Esenciales para la Humanidad S.A. de C.V. (Mexico City, Mexico) on January 2017, and its chemical/functional characterization and biosafety was evaluated (Supplementary File 1) before being used in this clinical trial.

### 4.1. Participants

Fifty-two sedentary (daily energy expenditure <4 metabolic equivalents (METs), as measured by a continuous physical activity questionnaire (IPAQ)) male adults, with a BMI over 25 kg·m^−2^ (27 overweight and 25 obese), volunteered to participate from May to September 2017. A detailed protocol for this trial has previously been registered at clinicaltrials.gov (https://clinicaltrials.gov/ct2/show/NCT02837666) and published [38]. For the recruitment of participants, an intra-school campaign and personalized interviews were conducted to ensure eligibility. The exclusion criteria of subjects were drinking more than 100 mL of alcohol a week, taking drugs and/or diet supplements, presenting a chronic disease, and having an impediment to practicing regular physical exercise. To follow the progress throughout the clinical phases of the study, the Consolidated Standards of Reporting Trials (CONSORT) checklist (Supplementary File 2) and flow diagram (Supplementary File 3) were followed. All participants were informed of the study purposes; physical, clinical, and biochemical procedures; and also about the risks associated with the physical intensity tests. Their acceptance was formalized by means of informed consent, and their confidentiality was strictly enforced. The Universidad Autónoma de Ciudad Juárez (UACJ) Review Board approved the study and all procedures (Reference number: CBE.ICB/062.09-15). All experimental procedures were conducted in accordance with the Declaration of Helsinki.

### 4.2. Baseline Measurements

Initially, each participant visited the exercise physiology laboratory at UACJ for baseline measurements. Body measurements were performed, with subjects lightly dressed and barefoot, using an electronic balance, and the standing height was measured with a stadiometer. Participants’ RHR was measured after five min of lying down in a bed. %BF was measured by plethysmography (BodPod, COSMED, Rome, Italy), according to the manufacturers’ guidelines. All measurements were repeated before each MIT.

### 4.3. Study Design

The clinical trial consisted of *S. maxima* (4.5 g·day^−1^) or placebo (4.5 g·day^−1^ of a low calorie saccharine powder) supplementation during 12 weeks in a randomized, double-blind, placebo-controlled, and counterbalanced crossover trial in a 2 × 2 factorial design. This experimental design was chosen to eliminate inter-individual differences related to *S. maxima*/placebo, and both supplements were encapsulated in dark capsules to mask their individual appearance and taste. The dose was chosen according to that reported in the literature in different *Spirulina* clinical trials, conducted to determine the biological effects of a washout period shorter than two weeks, to remove the effect of treatments and to avoid any possible delayed effect of *S. maxima* in the organism [14]. The exercise program duration accorded with the ACSM recommendations for the detection of decreases in BW, %BF, and improvements in cardiorespiratory fitness indicators [27].

**Figure 5:**
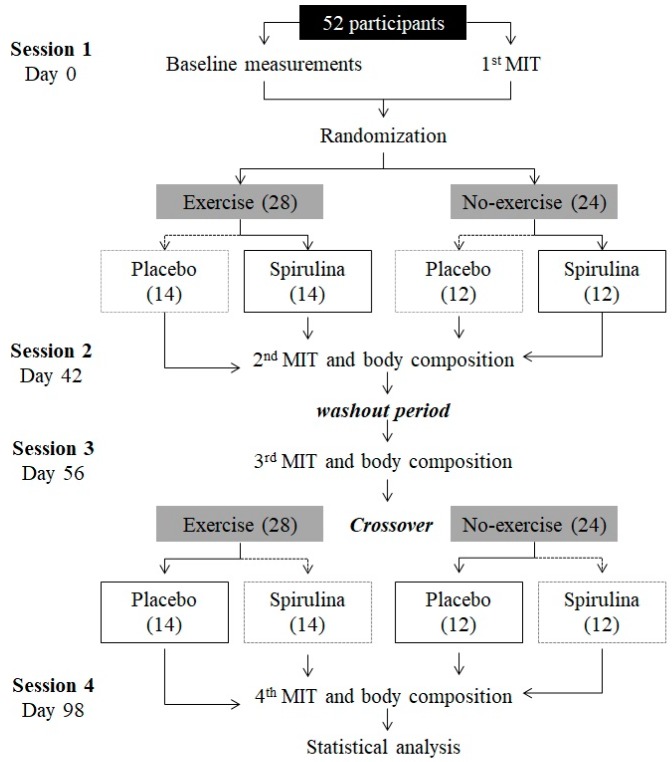
Experimental design for the independent and additive effect of *Spirulina maxima* and exercise. MIT: Maximum intensity test.

Eligible participants (*n* = 52) were randomly allocated to two of four combinations of two interventions (1. *S. maxima* supplementation, and 2. A systematic physical exercise program). These combinations included physical exercise (*n* = 28) with *S. maxima* (SE), a placebo (Ex), no-exercise (*n* = 24) with *S. maxima* (Sm), or placebo (C), as depicted in Figure 5. To assess compliance to the supplement intake, participants returned each week to the laboratory to receive new capsules. All treatment intake data were recorded on weekly case report forms.

The initial allocation was performed in such way that each group had almost the same proportion of overweight/obese (1:1) individuals using a computer-generated random schedule, stratified by a permuted block design because stratification in small trials confers an adequate balance and slightly more statistical power and precision [39]. Participants’ group allocations were performed by an independent researcher, who did not have any other participation during the study (double-blind). The sample size was determined by using the statistical program, G*Power [40], selecting a sample of >52 subjects, with α = 0.05 and power = 0.85.

The first period of treatment was carried out for six weeks, followed by a two-week wash-out period and, finally, a further six weeks of treatment for the second intervention groups (Figure 5).

### 4.4. Maximum Intensity Test (MIT)

Each subject participated in four stress tests performed at maximum intensity during the study. During MIT, consumed O_2_ and produced CO_2_ were obtained by a gas analyzer (Cortex MetaLyzer^®^ 3B, Leipzig, Germany), and HR was obtained using a Polar H7 sensor (Polar Electro, Lake Success, NY, USA). The anaerobic threshold was determined when OBLA reached 4 mM. The MIT protocol consisted of a cycle ergometer (Monark ergomedic 828 E; Monark exercise AB, 105 Vansbro, Sweden), initiated with a workload of 50–75 W, with increments of 15–30 W every 3 min for a minimum of 9 min and maximum of 15 min. At the end of each increment load, capillary blood samples were obtained to determine lactate using a YSI 1500 Sport Lactate Analyzer (YSI Life sciences, Yellow Springs, OH, USA). HR was recorded, and the physical perceived effort was registered using the Borg CR-10 scale. The anaerobic threshold was recorded through %HR at OBLA. According to the ACSM guidelines [25], the MIT was considered maximal if at least three of the following judgments were reached: failure of HR to increase with increases in workload, a peak respiratory exchange ratio ≥1.1, blood lactate concentration >8 mM, VO_2_ plateau (failure of VO_2_ to increase (150 mL·min^−1^) despite the increasing working rate), and rating of perceived exertion at peak exercise >7.

The first MIT was performed before beginning the first treatment, the day after the first supplementation period, and subjects returned to perform the second MIT, with identical conditions to the first one. The third MIT was performed after a wash-out period of two weeks to remove the effects of the treatment and avoid any possible delayed effect of *S. maxima* in the organism. The last MIT was performed after the second treatment, with identical conditions to the previous three times.

### 4.5. Dietary Analysis

All participants were subjected to a nutritional survey to define the daily calories required to establish dietary recommendations. Dietary intake was monitored by two trained nutritionists using three 24 h dietary recalls (semi-quantitative method), on days 0, 42, 56, and 98 (Figure 5), and two food frequency questionnaires (qualitative method), on days 0 and 98, according to Nelson’s guidelines [41], in order to ensure the independence of both dietary assessment tools. Dietary records were first inspected for missing data (e.g., missing food items or no complete responses) by an independent observer (nutritionist) and then analyzed for total calories, protein, carbohydrate, and fat intake (Diet Analysis Plus, ESHA Research, Salem, OR, USA). Lastly, compliance with the intake of supplements (*S. maxima* or placebo) and diet was monitored weekly by scheduled laboratory visits and carried out by trained nutritionists.

### 4.6. Physical Exercise Protocol

Exercise prescription was individual and accorded with the ACSM recommendations for persons with overweight and obesity [27]. Participants in the SE and Ex groups exercised for five days a week. The protocol began with a warm-up exercise (5–10 min), followed by muscular endurance exercise (20–30 min), then 20–30 min of cardiovascular exercise (walking, jogging, running, or cycling), and finally, five minutes of stretching. The intensity of cardiovascular exercise was administered as follows: For three of the five days, the intensity was between 50% and 80% of HR reserve, and for the other two days, the intensity was between 80% and 90% of HR reserve, using HIIT. Subjects performed the physical exercise program in the UACJ gym, under the technical supervision of a trainer.

### 4.7. Main Physiological Outcomes

The primary outcome of the study was the body composition response, because excess body fat is one of the major risk factors of CVD, and health organizations around the world recommend that studies focus on this response variable [1].

We included, as secondary endpoints, cardiorespiratory fitness variables, including TF, RHR, %HR at OBLA, and VO_2_max, since they have been associated with cardiovascular mortality in numerous epidemiologic studies, regardless of whether people presented any CVD [42].

### 4.8. Statistical Analysis

Data distribution normality was examined by the Shapiro–Wilk test, and the homoscedasticity was examined by the Levene test, for each group. Because the data of the studied variables did not present a normal distribution (*p* < 0.05), and the variances between groups were different, nonparametric analyses were performed. In order to analyze the differences between treatments, before and after the study, the Δ was analyzed by Kruskal–Wallis H and Mann–Whitney U tests (Supplementary File 4). To evaluate the associations among variables, a Spearman correlation was performed. The statistical significance was considered to be *p* < 0.05, using the software, SPSS 22.0 (SPSS Inc., Chicago, IL, USA), in all analyses.

## 5. Conclusions

*Spirulina maxima* intake, together with a systematic physical exercise program, has an individual and synergistic beneficial effect on body composition (decrease of BW and %BF) and cardiorespiratory parameters (an increase of TF, VO_2_max, OBLA, and a reduction of RHR) in overweight, but mostly in obese, male adults.

## Figures and Tables

**Table 1:** Anthropometric and physiological characteristics of participants

	Total	Overweight	Obesity
*N*	52	27	25
Age (y)	26 ± 5	26 ± 4	27 ± 6
Body weight (kg) *	90 ± 13	81 ± 8	100 ± 12
Height (m)	1.72 ± 0.1	1.72 ± 0.1	1.73 ± 0.1
BMI (kg·m^−2^) *	30.2 ± 4	27.4 ± 1.2	33.3 ± 3.8
Body fat percentage *	28.8 ± 7.2	24.8 ± 5.9	33.2 ± 6.1
Energy intake (kcal·day^−1^)	2054 ± 104	1977 ± 139	2054 ± 151
Maximal respiratory exchange ratio	1.19 ± 0	1.19 ± 0	1.18 ± 0.1
Resting HR (bpm)	64.4 ± 9.3	63.8 ± 6.9	65.2 ± 11.4
%HR at OBLA	61.9 ± 11.8	62.9 ± 11.5	60.8 ± 12.2
VO_2_max (mL·kg^−1^·min^−1^) *	35.4 ± 6.9	39.6 ± 5.1	30.8 ± 5.6

Data are expressed as mean ± SD. Asterisk (*) means statistical differences between overweight and obese individuals (*p* < 0.05); *N* = Sample size, BMI = body mass index, HR = heart rate, OBLA = onset blood lactate accumulation, and VO_2_max = maximal oxygen uptake.

**Table 2:** Correlation matrix among physical and physiological variables

	ΔBW	Δ%BF	ΔVO_2_max	ΔOBLA	ΔRHR
Δ%BF	0.33 **	1			
ΔVO_2_max	−0.54 **	−0.40 **	1		
ΔOBLA	−0.17	−0.14	0.11	1	
ΔRHR	0.34 **	0.06	−0.22 *	−0.04	1
ΔTF	−0.50 **	−0.19	0.36 **	0.16	−0.38 **

Δ = Differences between data obtained before and after the study, BW = body weight, %BF = percentage of body fat, VO_2_max = maximal oxygen uptake, OBLA = onset blood lactate accumulation, RHR = resting heart rate, and TF = time to fatigue. * *p* < 05, ** *p* < 0.01.

## Supplementary Materials

The following are available online at http://www.mdpi.com/1660-3397/16/10/364/s1: Supplementary File 1: Partial chemical characterization of *Spirulina maxima*; Supplementary File 2: CONSORT 2010 Checklist of information to include when reporting a randomized trial; Supplementary File 3: CONSORT 2010 Flow Diagram of the progress of the phases of the trial; and Supplementary File 4: Outputs of Mann Whitney U Tests (SPSS).
