# Supplementary material for: Effect of Arthrospira (Spirulina) maxima Supplementation and a Systematic Physical Exercise Program on the Body Composition and Cardiorespiratory Fitness of Overweight or Obese Subjects: A Double-Blind, Randomized, and Crossover Controlled Trial
Source: Mar Drugs. 2018 Oct 1;16(10):364. doi: 10.3390/md16100364 (PMC6213464; doi:10.3390/md16100364)
Supplement: Supplementary file 1 [file marinedrugs-16-00364-s001.zip › Supplementary Files/Supplementary File 1.pdf]

Supplementary File 1 - Partial chemical characterization of *Spirulina maxima*

Methods

1. *Microbiological analysis*. Total aerobic mesophilic bacteria, total coliforms, molds and yeast were determined by plating diluted spirulina samples in 3M™ Petrifilm™ plates, following manufacturing instructions.
2. *Heavy metals*. Chromium, zinc, lead, cadmium and arsenic (ppm) were analyzed according recommendations from the United States Protection Agency [1], by atomic absorption spectrometry (Perkín Elmer Analist 200, USA).
3. *Chemical analysis*. Protein, fat, moisture, ash and carbohydrates (by difference) were assayed by approved AOAC methods procedures by triplicate.
4. *SDS-PAGE*. Molecular distribution of reduced/denatured protein species in S. *maxima* was determined by sodium dodecyl sulfate (SDS) gel electrophoresis according to Laemmli [2].
5. *Antioxidant profile*. Total phenolic compounds were determined by the method reported by García-Galaz *et al.* [3], total flavonoids by the method reported by Álvarez-Parrilla *et al.* [4], and the total carotenoids/chlorophylls according to Jensen [5].

Results

1. *S. maxima* showed absence of coliforms, yeasts, molds and mesophilic aerobes presence, so it was considered safe for human consumption.
2. The content (ppm) of Pb (0.11 ± 0.02), Cd (0.002 ± 0.003), As (0.04 ± 0.05), Zn (1.54 ± 0.18) in *S. maxima* were within the permissible limits.
3. S. *maxima* showed a total of 65% protein, 9% ash, 9% moisture, 1% lipids and 16% carbohydrates (by difference).
4. SDS-PAGE allowed the detection of two major proteins with and average molecular weight of 18 and 21 kDa (**Figure S1**), perhaps corresponding to Chethana *et al* [6].
5. Major antioxidant phytochemical content in *S. maxima* is described in **Table S1**.

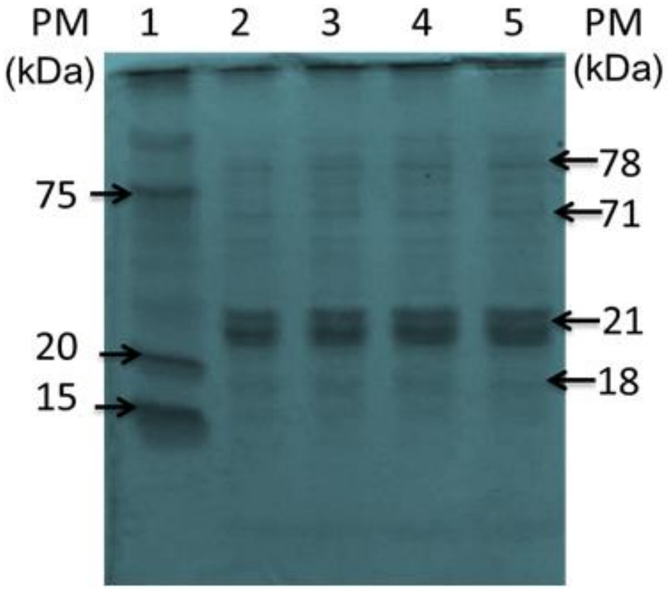

Figure S1. SDS-PAGE of *S. maxima*. Lane 1= Molecular marker; Lanes 2,3= *S. maxima* sample (10 µg/mL); Lanes 4,5= *S. maxima* sample (15 µg/mL).

Table S1. Antioxidant phytochemicals in *Spirulina maxima*

Total phenolics mgGAE/g DW	Flavonoids mgCE/g DW	Carotenoids mg β-CE/g DW	Chlorophylls mg/g DW
4.9 ± 0.22	0.9 ± 0.06	17.1 ± 0.37	41.9 ± 1.32

Dry weight (DW); gallic acid (GAE), catechin (CE) and β-carotene (β-CE) equivalents

References

1. United States Enviromental Protection Agency (USEPA). *Methods for the Determinations of Metals in Environmental Samples*; EPA-600/4-91-010; USEPA: Cincinnati, OH, USA, 1991.
2. Laemmli, U.K. Cleavage of structural proteins during the assembly of the head of bacteriophage T4. *Nature* **1970**, *227*, 680–685.
3. Garcia-Galaz, A.; Gutiérrez-Millán, L.E.; Valdés-Covarrubias, M.A.; Acedo-Félix, E.; Burgos-Hernández, A.; López-Torres, M.A.; Burboa-Zazueta, M.G. Antiproliferative and antibacterial activity evaluation of red microalgae Rhodosorus marinus. *Afr. J. Biotechnol.* **2014**, *13*, 4169–4175, doi:10.5897/AJB2014.13943.
4. Álvarez-Parrilla, E.; Mercado-Mercado, G.; de la Rosa, L.A.; López-Díaz, J.A.; Wall-Medrano, A.; González-Aguilar, G.A. Antioxidant activity and prevention of pork meat lipid oxidation using traditional Mexican condiments (pasilla dry pepper, achiote, and mole sauce). *Food Sci. Technol.* **2014**, *34*, 371–378, doi:10.1590/fst.2014.0052.
5. Jensen, A. Chlorophyll and carotenoids. In *Handbook of physiochemical and biochemical methods*; Hallebust, J.A., Craigie, J.S., Eds.; Cambridge University Press: Cambridge, UK, 1978; pp. 5–70.
6. Chethana, S.; Nayak, C.A.; Madhusudhan, M.C.; Raghavarao, K.S.M.S. Single step aqueous two-phase extraction for downstream processing of C-phycocyanin from *Spirulina platensis*. *J. Food Sci. Technol.* **2015**, *52*, 2415–2421, doi:10.1007/s13197-014-1287-9.
