# Supplementary material for: Effect of Arthrospira (Spirulina) maxima Supplementation and a Systematic Physical Exercise Program on the Body Composition and Cardiorespiratory Fitness of Overweight or Obese Subjects: A Double-Blind, Randomized, and Crossover Controlled Trial
Source: Mar Drugs. 2018 Oct 1;16(10):364. doi: 10.3390/md16100364 (PMC6213464; doi:10.3390/md16100364)
Supplement: Supplementary file 1 [file marinedrugs-16-00364-s001.zip › Supplementary Files/Supplementary File 2.pdf]

Supplementary File 2. CONSORT 2010 Checklist of information to include when reporting a randomized trial

Section/Topic	Item No	Checklist item	Reported on page No
**Title and abstract**			
	1a	Identification as a randomised trial in the title	Page 1
	1b	Structured summary of trial design, methods, results, and conclusions (for specific guidance see CONSORT for abstracts)	Page 1
**Introduction**			
Background and objectives	2a	Scientific background and explanation of rationale	Pages 1-2
	2b	Specific objectives or hypotheses	Pages 2,10
**Methods**			
Trial design	3a	Description of trial design (such as parallel, factorial) including allocation ratio	Page 9
	3b	Important changes to methods after trial commencement (such as eligibility criteria), with reasons	N/A
Participants	4a	Eligibility criteria for participants	Page 8
	4b	Settings and locations where the data were collected	Page 4
Interventions	5	The interventions for each group with sufficient details to allow replication, including how and when they were actually administered	Pages 9-10
Outcomes	6a	Completely defined pre-specified primary and secondary outcome measures, including how and when they were assessed	Pages 9-10
	6b	Any changes to trial outcomes after the trial commenced, with reasons	N/A
Sample size	7a	How sample size was determined	Page 9
	7b	When applicable, explanation of any interim analyses and stopping guidelines	N/A
Randomisation:			
Sequence generation	8a	Method used to generate the random allocation sequence	Page 9
	8b	Type of randomisation; details of any restriction (such as blocking and block size)	Page 9
Allocation concealment mechanism	9	Mechanism used to implement the random allocation sequence (such as sequentially numbered containers), describing any steps taken to conceal the sequence until interventions were assigned	Page 9
Implementation	10	Who generated the random allocation sequence, who enrolled participants, and who assigned participants to interventions	Page 9
Blinding	11a	If done, who was blinded after assignment to interventions (for example, participants, care providers, those assessing outcomes) and how	Page 9

	11b	If relevant, description of the similarity of interventions	N/A
Statistical methods	12a	Statistical methods used to compare groups for primary and secondary outcomes	Page 11
	12b	Methods for additional analyses, such as subgroup analyses and adjusted analyses	Page 11
**Results**			
Participant flow (a diagram is strongly recommended)	13a	For each group, the numbers of participants who were randomly assigned, received intended treatment, and were analysed for the primary outcome	Figure 5
	13b	For each group, losses and exclusions after randomisation, together with reasons	N/A
Recruitment	14a	Dates defining the periods of recruitment and follow-up	Page 8
	14b	Why the trial ended or was stopped	N/A
Baseline data	15	A table showing baseline demographic and clinical characteristics for each group	Table 1
Numbers analysed	16	For each group, number of participants (denominator) included in each analysis and whether the analysis was by original assigned groups	Figure 5
Outcomes and estimation	17a	For each primary and secondary outcome, results for each group, and the estimated effect size and its precision (such as 95% confidence interval)	Pages 2-5
	17b	For binary outcomes, presentation of both absolute and relative effect sizes is recommended	N/A
Ancillary analyses	18	Results of any other analyses performed, including subgroup analyses and adjusted analyses, distinguishing pre-specified from exploratory	N/A
Harms	19	All important harms or unintended effects in each group (for specific guidance see CONSORT for harms)	N/A
**Discussion**			
Limitations	20	Trial limitations, addressing sources of potential bias, imprecision, and, if relevant, multiplicity of analyses	N/A
Generalisability	21	Generalisability (external validity, applicability) of the trial findings	Pages 6-8
Interpretation	22	Interpretation consistent with results, balancing benefits and harms, and considering other relevant evidence	Pages 6-8
**Other information**			
Registration	23	Registration number and name of trial registry	Page 8
Protocol	24	Where the full trial protocol can be accessed, if available	Page 1
Funding	25	Sources of funding and other support (such as supply of drugs), role of funders	Page 11
