# Supplementary material for: Effect of Arthrospira (Spirulina) maxima Supplementation and a Systematic Physical Exercise Program on the Body Composition and Cardiorespiratory Fitness of Overweight or Obese Subjects: A Double-Blind, Randomized, and Crossover Controlled Trial
Source: Mar Drugs. 2018 Oct 1;16(10):364. doi: 10.3390/md16100364 (PMC6213464; doi:10.3390/md16100364)

CONSORT
TRANSPARENT REPORTING of TRIALS

Supplementary File 3. CONSORT 2010 Flow Diagram of the progress through the phases of the trial.

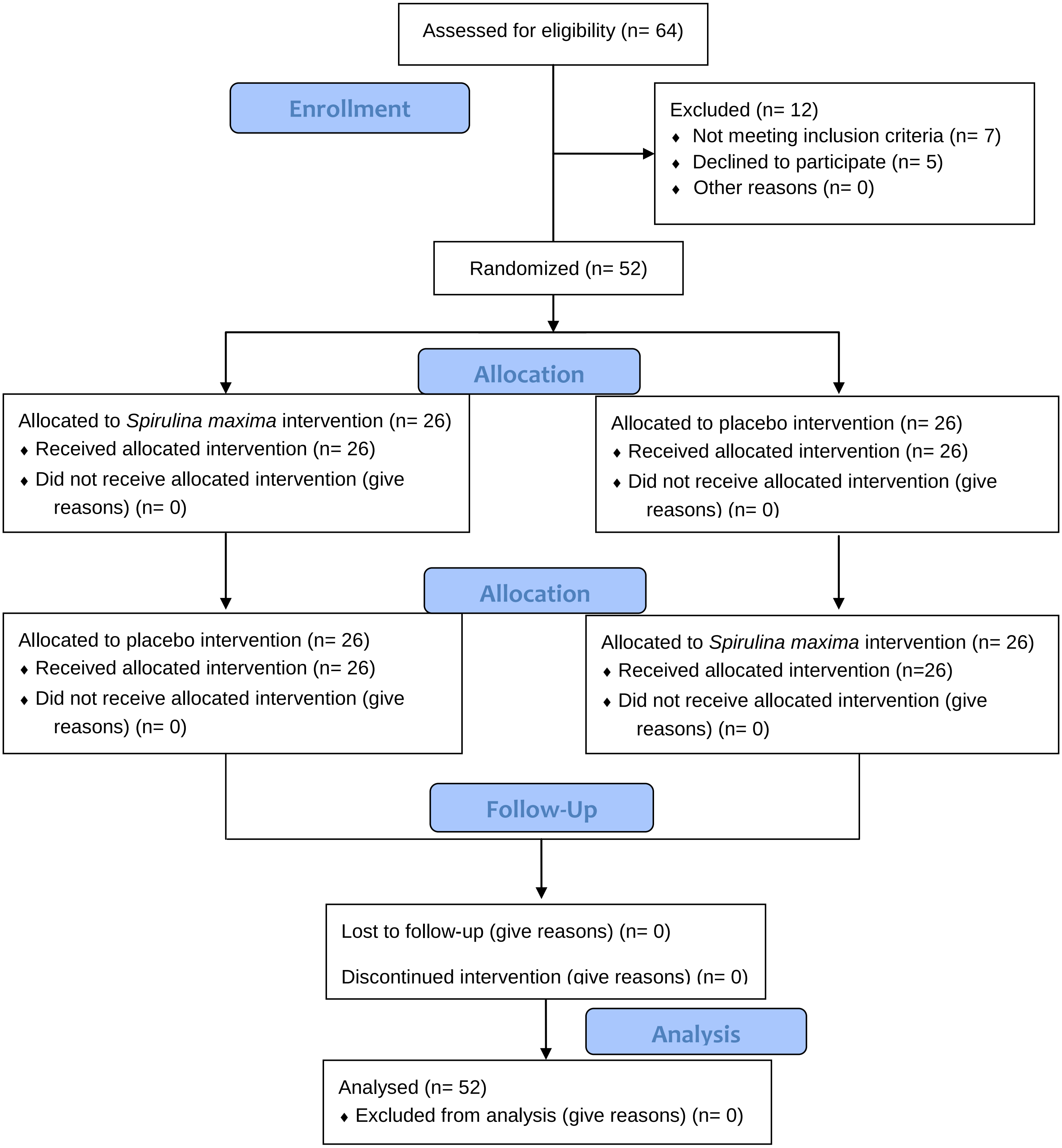

Supplement: Supplementary file 1 [file marinedrugs-16-00364-s001.zip › Supplementary Files/Supplementary File 3.pdf]
