# Supplementary material for: Effect of Arthrospira (Spirulina) maxima Supplementation and a Systematic Physical Exercise Program on the Body Composition and Cardiorespiratory Fitness of Overweight or Obese Subjects: A Double-Blind, Randomized, and Crossover Controlled Trial
Source: Mar Drugs. 2018 Oct 1;16(10):364. doi: 10.3390/md16100364 (PMC6213464; doi:10.3390/md16100364)
Supplement: Supplementary file 1 [file marinedrugs-16-00364-s001.zip › Supplementary Files/Supplementary File 4.pdf]

Supplementary File 4 - Outputs of Mann Whitney U Tests (SPSS)

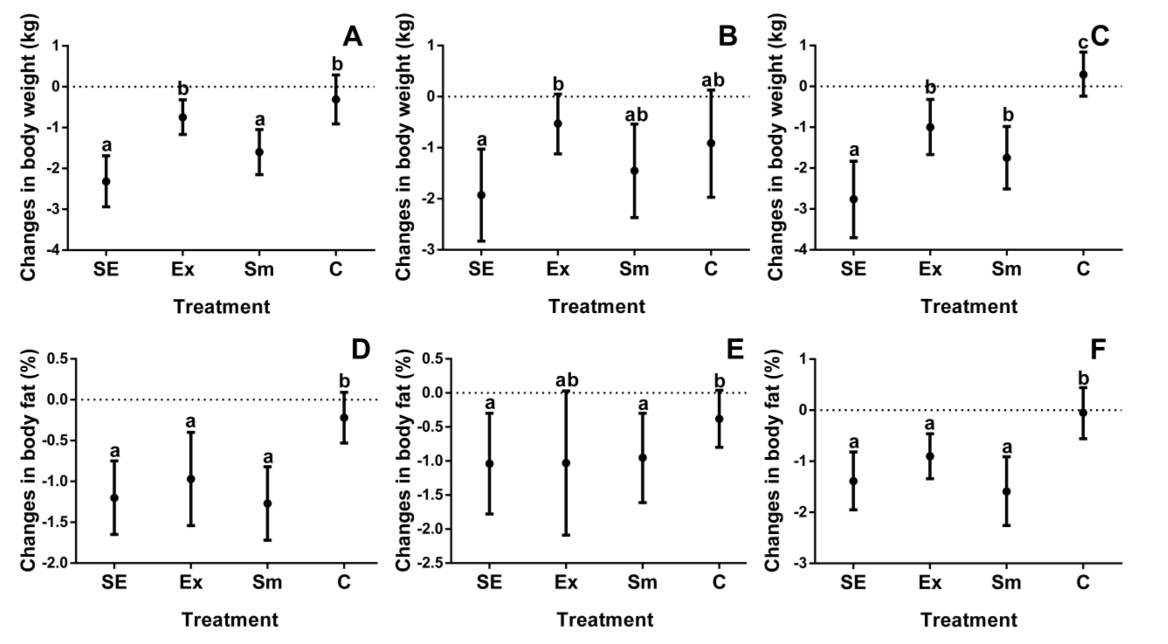

Figure 1. Changes in body weight and body fat percentage by treatments. SE: *Spirulina* and exercise; Ex: Exercise and placebo; Sm: *Spirulina* without exercise; and C: Control (Placebo treatment). (**A**) Total body weight changes of subjects; (**B**) Body weight changes in overweight subjects; (**C**) Body weight changes in subjects with obesity; (**D**) Total body fat changes of subjects; (**E**) Body fat changes in overweight subjects; and (**F**) Body fat changes in subjects with obesity. Data are shown as the mean, with 95% confidence intervals. Different letters indicate the statistical differences between groups ($p < 0.05$).

Figure 1A

SE vs Ex

Test Statistics[a]

	Changes_BW
Mann-Whitney U	152.500
Wilcoxon W	558.500
Z	-4.020
Asymp. Sig. (2-tailed)	.000

a. Grouping Variable: Group_TOTAL

SE vs Sm

Test Statistics[a]

	Changes_BW
Mann-Whitney U	242.500
Wilcoxon W	648.500
Z	-1.769
Asymp. Sig. (2-tailed)	.077

a. Grouping Variable: Group_TOTAL

SE vs C

Test Statistics[a]

	Changes_BW
Mann-Whitney U	96.000
Wilcoxon W	502.000
Z	-4.464
Asymp. Sig. (2-tailed)	.000

a. Grouping Variable: Group_TOTAL

Ex vs Sm

Test Statistics[a]

	Changes_BW
Mann-Whitney U	207.000
Wilcoxon W	507.000
Z	-2.438
Asymp. Sig. (2-tailed)	.015

a. Grouping Variable: Group_TOTAL

Ex vs C

Test Statistics[a]

	Changes_BW
Mann-Whitney U	244.000
Wilcoxon W	650.000
Z	-1.729
Asymp. Sig. (2-tailed)	.084

a. Grouping Variable: Group_TOTAL

Sm vs C

Test Statistics[a]

	Changes_BW
Mann-Whitney U	126.500
Wilcoxon W	426.500
Z	-3.379
Asymp. Sig. (2-tailed)	.001

a. Grouping Variable: Group_TOTAL

Figure 1B

SE vs Ex

Test Statistics[a]

	Changes_BW
Mann-Whitney U	55.000
Wilcoxon W	175.000
Z	-2.477
Asymp. Sig. (2-tailed)	.013
Exact Sig. [2*(1-tailed Sig.)]	.016[b]

a. Grouping Variable: Group_OW_Ob

b. Not corrected for ties.

SE vs Sm

Test Statistics[a]

	Changes_BW
Mann-Whitney U	76.000
Wilcoxon W	196.000
Z	-.699
Asymp. Sig. (2-tailed)	.484
Exact Sig. [2*(1-tailed Sig.)]	.516[b]

a. Grouping Variable: Group_OW_Ob

b. Not corrected for ties.

SE vs C

Test Statistics[a]

	Changes_BW
Mann-Whitney U	58.000
Wilcoxon W	178.000
Z	-1.597
Asymp. Sig. (2-tailed)	.110
Exact Sig. [2*(1-tailed Sig.)]	.126[b]

a. Grouping Variable: Group_OW_Ob

b. Not corrected for ties.

Ex vs Sm

Test Statistics[a]

	Changes_BW
Mann-Whitney U	56.000
Wilcoxon W	134.000
Z	-1.722
Asymp. Sig. (2-tailed)	.085
Exact Sig. [2*(1-tailed Sig.)]	.103[b]

a. Grouping Variable: Group_OW_Ob

b. Not corrected for ties.

Ex vs C

Test Statistics[a]

	Changes_BW
Mann-Whitney U	78.500
Wilcoxon W	156.500
Z	-.584
Asymp. Sig. (2-tailed)	.559
Exact Sig. [2*(1-tailed Sig.)]	.581[b]

a. Grouping Variable: Group_OW_Ob

b. Not corrected for ties.

Sm vs C

Test Statistics[a]

	Changes_BW
Mann-Whitney U	53.500
Wilcoxon W	131.500
Z	-1.090
Asymp. Sig. (2-tailed)	.276
Exact Sig. [2*(1-tailed Sig.)]	.291[b]

a. Grouping Variable: Group_OW_Ob

b. Not corrected for ties.

Figure 1C

SE vs Ex

Test Statistics[a]

	Changes_BW
Mann-Whitney U	19.500
Wilcoxon W	110.500
Z	-3.422
Asymp. Sig. (2-tailed)	.001
Exact Sig. [2*(1-tailed Sig.)]	.000[b]

a. Grouping Variable: Group_OW_Ob

b. Not corrected for ties.

SE vs Sm

Test Statistics[a]

	Changes_BW
Mann-Whitney U	40.000
Wilcoxon W	131.000
Z	-2.172
Asymp. Sig. (2-tailed)	.030
Exact Sig. [2*(1-tailed Sig.)]	.040[b]

a. Grouping Variable: Group_OW_Ob

b. Not corrected for ties.

SE vs C

Test Statistics[a]

	Changes_BW
Mann-Whitney U	.500
Wilcoxon W	91.500
Z	-4.299
Asymp. Sig. (2-tailed)	.000
Exact Sig. [2*(1-tailed Sig.)]	.000[b]

a. Grouping Variable: Group_OW_Ob

b. Not corrected for ties.

Ex vs Sm

Test Statistics[a]

	Changes_BW
Mann-Whitney U	50.000
Wilcoxon W	128.000
Z	-1.561
Asymp. Sig. (2-tailed)	.119
Exact Sig. [2*(1-tailed Sig.)]	.137[b]

a. Grouping Variable: Group_OW_Ob

b. Not corrected for ties.

Ex vs C

Test Statistics[a]

	Changes_BW
Mann-Whitney U	26.500
Wilcoxon W	117.500
Z	-2.852
Asymp. Sig. (2-tailed)	.004
Exact Sig. [2*(1-tailed Sig.)]	.004[b]

a. Grouping Variable: Group_OW_Ob

b. Not corrected for ties.

Sm vs C

Test Statistics[a]

	Changes_BW
Mann-Whitney U	7.000
Wilcoxon W	85.000
Z	-3.795
Asymp. Sig. (2-tailed)	.000
Exact Sig. [2*(1-tailed Sig.)]	.000[b]

a. Grouping Variable: Group_OW_Ob

b. Not corrected for ties.

Figure 1D

SE vs Ex

Test Statistics[a]

	Changes_BF
Mann-Whitney U	283,500
Wilcoxon W	689,500
Z	-1,780
Asymp. Sig. (2-tailed)	,075

a. Grouping Variable: Group_TOTAL

Ex vs Sm

Test Statistics[a]

	Changes_BF
Mann-Whitney U	232,000
Wilcoxon W	532,000
Z	-1,912
Asymp. Sig. (2-tailed)	,056

a. Grouping Variable: Group_TOTAL

SE vs Sm

Test Statistics[a]

	Changes_BF
Mann-Whitney U	325,500
Wilcoxon W	625,500
Z	-,193
Asymp. Sig. (2-tailed)	,847

a. Grouping Variable: Group_TOTAL

Ex vs C

Test Statistics[a]

	Changes_BF
Mann-Whitney U	172,000
Wilcoxon W	578,000
Z	-3,016
Asymp. Sig. (2-tailed)	,003

a. Grouping Variable: Group_TOTAL

SE vs C

Test Statistics[a]

	Changes_BF
Mann-Whitney U	138,000
Wilcoxon W	544,000
Z	-3,640
Asymp. Sig. (2-tailed)	,000

a. Grouping Variable: Group_TOTAL

Sm vs C

Test Statistics[a]

	Changes_BF
Mann-Whitney U	100,500
Wilcoxon W	400,500
Z	-3,873
Asymp. Sig. (2-tailed)	,000

a. Grouping Variable: Group_TOTAL

Figure 1E

SE vs Ex

Test Statistics[a]

	Changes_BF
Mann-Whitney U	87,000
Wilcoxon W	207,000
Z	-1,059
Asymp. Sig. (2-tailed)	,290
Exact Sig. [2*(1-tailed Sig.)]	,305[b]

a. Grouping Variable: Group_OW_Ob

b. Not corrected for ties.

Ex vs Sm

Test Statistics[a]

	Changes_BF
Mann-Whitney U	64,500
Wilcoxon W	142,500
Z	-1,247
Asymp. Sig. (2-tailed)	,212
Exact Sig. [2*(1-tailed Sig.)]	,217[b]

a. Grouping Variable: Group_OW_Ob

b. Not corrected for ties.

SE vs Sm

Test Statistics[a]

	Changes_BF
Mann-Whitney U	85,500
Wilcoxon W	205,500
Z	-,220
Asymp. Sig. (2-tailed)	,826
Exact Sig. [2*(1-tailed Sig.)]	,829[b]

a. Grouping Variable: Group_OW_Ob

b. Not corrected for ties.

Ex vs C

Test Statistics[a]

	Changes_BF
Mann-Whitney U	60,500
Wilcoxon W	180,500
Z	-1,444
Asymp. Sig. (2-tailed)	,149
Exact Sig. [2*(1-tailed Sig.)]	,152[b]

a. Grouping Variable: Group_OW_Ob

b. Not corrected for ties.

SE vs C

Test Statistics[a]

	Changes_BF
Mann-Whitney U	48,000
Wilcoxon W	168,000
Z	-2,052
Asymp. Sig. (2-tailed)	,040
Exact Sig. [2*(1-tailed Sig.)]	,041[b]

a. Grouping Variable: Group_OW_Ob

b. Not corrected for ties.

Sm vs C

Test Statistics[a]

	Changes_BF
Mann-Whitney U	36,500
Wilcoxon W	114,500
Z	-2,054
Asymp. Sig. (2-tailed)	,040
Exact Sig. [2*(1-tailed Sig.)]	,039[b]

a. Grouping Variable: Group_OW_Ob

b. Not corrected for ties.

Figure 1F

SE vs Ex

Test Statistics[a]

	Changes_BF
Mann-Whitney U	60,500
Wilcoxon W	151,500
Z	-1,233
Asymp. Sig. (2-tailed)	,217
Exact Sig. [2*(1-tailed Sig.)]	,223[b]

a. Grouping Variable: Group_OW_Ob

b. Not corrected for ties.

SE vs Sm

Test Statistics[a]

	Changes_BF
Mann-Whitney U	66,500
Wilcoxon W	144,500
Z	-,627
Asymp. Sig. (2-tailed)	,531
Exact Sig. [2*(1-tailed Sig.)]	,538[b]

a. Grouping Variable: Group_OW_Ob

b. Not corrected for ties.

SE vs C

Test Statistics[a]

	Changes_BF
Mann-Whitney U	19,500
Wilcoxon W	110,500
Z	-3,189
Asymp. Sig. (2-tailed)	,001
Exact Sig. [2*(1-tailed Sig.)]	,001[b]

a. Grouping Variable: Group_OW_Ob

b. Not corrected for ties.

Ex vs Sm

Test Statistics[a]

	Changes_BF
Mann-Whitney U	49,000
Wilcoxon W	127,000
Z	-1,581
Asymp. Sig. (2-tailed)	,114
Exact Sig. [2*(1-tailed Sig.)]	,123[b]

a. Grouping Variable: Group_OW_Ob

b. Not corrected for ties.

Ex vs C

Test Statistics[a]

	Changes_BF
Mann-Whitney U	26,500
Wilcoxon W	117,500
Z	-2,808
Asymp. Sig. (2-tailed)	,005
Exact Sig. [2*(1-tailed Sig.)]	,004[b]

a. Grouping Variable: Group_OW_Ob

b. Not corrected for ties.

Sm vs C

Test Statistics[a]

	Changes_BF
Mann-Whitney U	13,000
Wilcoxon W	91,000
Z	-3,417
Asymp. Sig. (2-tailed)	,001
Exact Sig. [2*(1-tailed Sig.)]	,000[b]

a. Grouping Variable: Group_OW_Ob

b. Not corrected for ties.

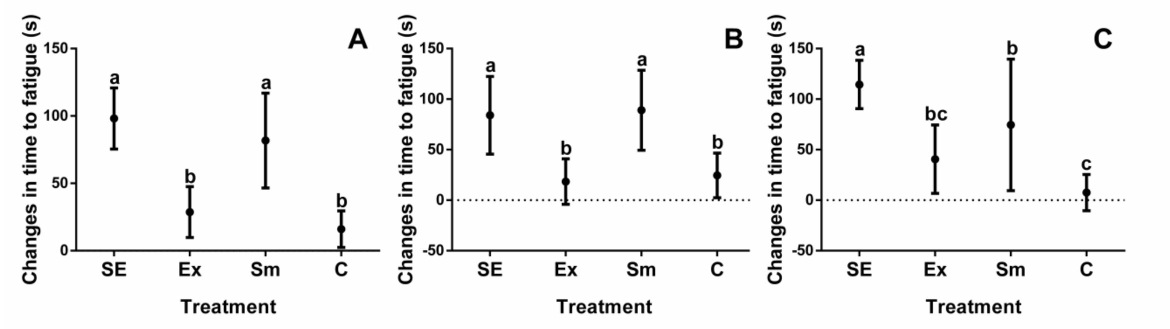

Figure 2. Changes in time to fatigue by treatments. SE: *Spirulina* and exercise; Ex: Exercise and placebo; Sm: *Spirulina* without exercise; and C: Control (Placebo treatment). (**A**) Total time to fatigue changes of subjects; (**B**) Time to fatigue changes in overweight subjects; and (**C**) Time to fatigue changes in obesity subjects. Data are shown as the mean, with 95% confidence intervals. Different letters indicate the statistical differences between groups ($p < 0.05$).

Figure 2A

SE vs Ex

Test Statistics[a]

	Changes_TF
Mann-Whitney U	146,500
Wilcoxon W	552,500
Z	-4,040
Asymp. Sig. (2-tailed)	,000

a. Grouping Variable: Group_TOTAL

Ex vs Sm

Test Statistics[a]

	Changes_TF
Mann-Whitney U	201,000
Wilcoxon W	607,000
Z	-2,510
Asymp. Sig. (2-tailed)	,012

a. Grouping Variable: Group_TOTAL

SE vs Sm

Test Statistics[a]

	Changes_TF
Mann-Whitney U	256,500
Wilcoxon W	556,500
Z	-1,464
Asymp. Sig. (2-tailed)	,143

a. Grouping Variable: Group_TOTAL

Ex vs C

Test Statistics[a]

	Changes_TF
Mann-Whitney U	273,500
Wilcoxon W	573,500
Z	-1,208
Asymp. Sig. (2-tailed)	,227

a. Grouping Variable: Group_TOTAL

SE vs C

Test Statistics[a]

	Changes_TF
Mann-Whitney U	72,500
Wilcoxon W	372,500
Z	-4,926
Asymp. Sig. (2-tailed)	,000

a. Grouping Variable: Group_TOTAL

Sm vs C

Test Statistics[a]

	Changes_TF
Mann-Whitney U	122,500
Wilcoxon W	422,500
Z	-3,566
Asymp. Sig. (2-tailed)	,000

a. Grouping Variable: Group_TOTAL

Figure 2B

SE vs Ex

Test Statistics[a]

	Changes_TF
Mann-Whitney U	47,500
Wilcoxon W	167,500
Z	-2,734
Asymp. Sig. (2-tailed)	,006
Exact Sig. [2*(1-tailed Sig.)]	,006[b]

a. Grouping Variable: Group_OW_Ob

b. Not corrected for ties.

Ex vs Sm

Test Statistics[a]

	Changes_TF
Mann-Whitney U	31,500
Wilcoxon W	151,500
Z	-2,894
Asymp. Sig. (2-tailed)	,004
Exact Sig. [2*(1-tailed Sig.)]	,003[b]

a. Grouping Variable: Group_OW_Ob

b. Not corrected for ties.

SE vs Sm

Test Statistics[a]

	Changes_TF
Mann-Whitney U	84,000
Wilcoxon W	204,000
Z	-,294
Asymp. Sig. (2-tailed)	,769
Exact Sig. [2*(1-tailed Sig.)]	,792[b]

a. Grouping Variable: Group_OW_Ob

b. Not corrected for ties.

Ex vs C

Test Statistics[a]

	Changes_TF
Mann-Whitney U	81,500
Wilcoxon W	201,500
Z	-,447
Asymp. Sig. (2-tailed)	,655
Exact Sig. [2*(1-tailed Sig.)]	,683[b]

a. Grouping Variable: Group_OW_Ob

b. Not corrected for ties.

SE vs C

Test Statistics[a]

	Changes_TF
Mann-Whitney U	39,500
Wilcoxon W	117,500
Z	-2,513
Asymp. Sig. (2-tailed)	,012
Exact Sig. [2*(1-tailed Sig.)]	,012[b]

a. Grouping Variable: Group_OW_Ob

b. Not corrected for ties.

Sm vs C

Test Statistics[a]

	Changes_TF
Mann-Whitney U	26,500
Wilcoxon W	104,500
Z	-2,678
Asymp. Sig. (2-tailed)	,007
Exact Sig. [2*(1-tailed Sig.)]	,007[b]

a. Grouping Variable: Group_OW_Ob

b. Not corrected for ties.

Figure 2C

SE vs Ex

Test Statistics[a]

	Changes_TF
Mann-Whitney U	24,500
Wilcoxon W	115,500
Z	-3,079
Asymp. Sig. (2-tailed)	,002
Exact Sig. [2*(1-tailed Sig.)]	,001[b]

a. Grouping Variable: Group_OW_Ob

b. Not corrected for ties.

Ex vs Sm

Test Statistics[a]

	Changes_TF
Mann-Whitney U	70,000
Wilcoxon W	161,000
Z	-,440
Asymp. Sig. (2-tailed)	,660
Exact Sig. [2*(1-tailed Sig.)]	,689[b]

a. Grouping Variable: Group_OW_Ob

b. Not corrected for ties.

SE vs Sm

Test Statistics[a]

	Changes_TF
Mann-Whitney U	37,500
Wilcoxon W	115,500
Z	-2,214
Asymp. Sig. (2-tailed)	,027
Exact Sig. [2*(1-tailed Sig.)]	,026[b]

a. Grouping Variable: Group_OW_Ob

b. Not corrected for ties.

Ex vs C

Test Statistics[a]

	Changes_TF
Mann-Whitney U	43,500
Wilcoxon W	121,500
Z	-1,939
Asymp. Sig. (2-tailed)	,052
Exact Sig. [2*(1-tailed Sig.)]	,060[b]

a. Grouping Variable: Group_OW_Ob

b. Not corrected for ties.

SE vs C

Test Statistics[a]

	Changes_TF
Mann-Whitney U	4,000
Wilcoxon W	82,000
Z	-4,093
Asymp. Sig. (2-tailed)	,000
Exact Sig. [2*(1-tailed Sig.)]	,000[b]

a. Grouping Variable: Group_OW_Ob

b. Not corrected for ties.

Sm vs C

Test Statistics[a]

	Changes_TF
Mann-Whitney U	34,000
Wilcoxon W	112,000
Z	-2,392
Asymp. Sig. (2-tailed)	,017
Exact Sig. [2*(1-tailed Sig.)]	,028[b]

a. Grouping Variable: Group_OW_Ob

b. Not corrected for ties.

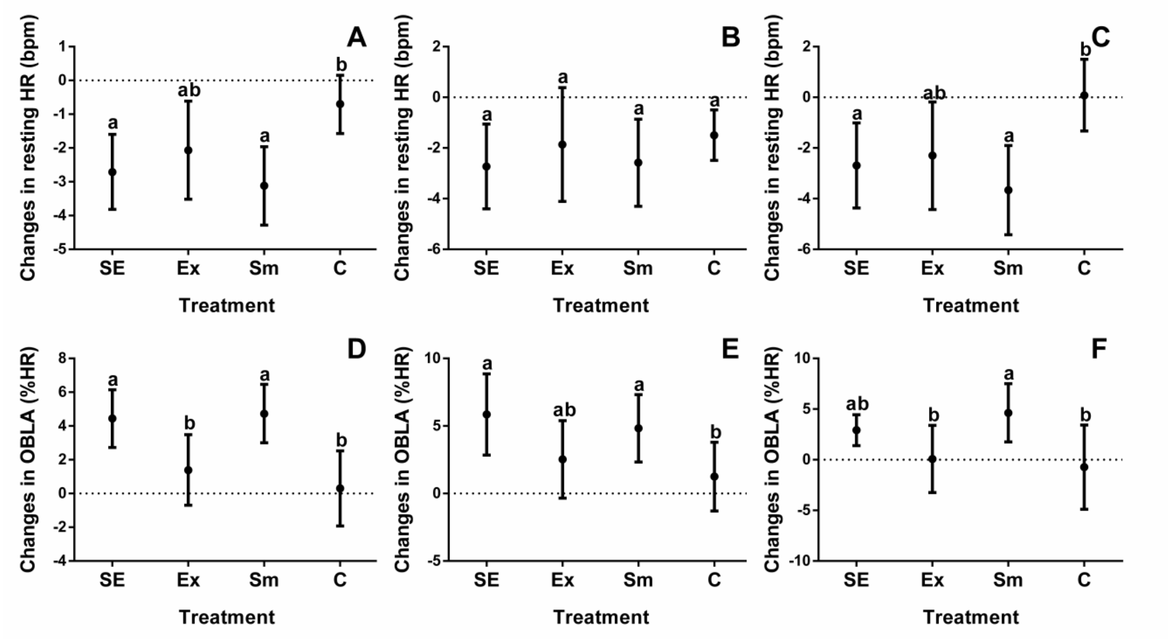

Figure 3. Changes in resting heart rate and onset blood lactate accumulation by treatments. HR: Heart rate; OBLA: Onset blood lactate accumulation; %HR: Percentage of maximal heart rate; SE: *Spirulina* and exercise; Ex: Exercise and placebo; Sm: *Spirulina* without exercise; and C: Control (Placebo treatment). (**A**) Total resting HR changes of subjects; (**B**) Resting HR changes in overweight subjects; (**C**) Resting HR changes in obesity subjects; (**D**) Total OBLA changes of subjects; (**E**) OBLA changes in overweight subjects; and (**F**) OBLA changes in obesity subjects. Data are presented as the mean with 95% confidence intervals. Different letters indicate the statistical differences between groups ($p < 0.05$).

Figure 3A

SE vs Ex

Test Statistics[a]

	Changes_HR
Mann-Whitney U	320,000
Wilcoxon W	726,000
Z	-1,188
Asymp. Sig. (2-tailed)	,235

a. Grouping Variable: Group_TOTAL

Ex vs Sm

Test Statistics[a]

	Changes_HR
Mann-Whitney U	248,000
Wilcoxon W	548,000
Z	-1,625
Asymp. Sig. (2-tailed)	,104

a. Grouping Variable: Group_TOTAL

SE vs Sm

Test Statistics[a]

	Changes_HR
Mann-Whitney U	303,500
Wilcoxon W	603,500
Z	-,601
Asymp. Sig. (2-tailed)	,548

a. Grouping Variable: Group_TOTAL

Ex vs C

Test Statistics[a]

	Changes_HR
Mann-Whitney U	274,000
Wilcoxon W	680,000
Z	-1,163
Asymp. Sig. (2-tailed)	,245

a. Grouping Variable: Group_TOTAL

SE vs C

Test Statistics[a]

	Changes_HR
Mann-Whitney U	176,000
Wilcoxon W	582,000
Z	-3,000
Asymp. Sig. (2-tailed)	,003

a. Grouping Variable: Group_TOTAL

Sm vs C

Test Statistics[a]

	Changes_HR
Mann-Whitney U	130,000
Wilcoxon W	430,000
Z	-3,305
Asymp. Sig. (2-tailed)	,001

a. Grouping Variable: Group_TOTAL

Figure 3B

SE vs Ex

Test Statistics[a]

	Changes_HR
Mann-Whitney U	88,500
Wilcoxon W	208,500
Z	-1,003
Asymp. Sig. (2-tailed)	,316
Exact Sig. [2*(1-tailed Sig.)]	,325[b]

a. Grouping Variable: Group_OW_Ob

b. Not corrected for ties.

SE vs Sm

Test Statistics[a]

	Changes_HR
Mann-Whitney U	88,500
Wilcoxon W	208,500
Z	-,074
Asymp. Sig. (2-tailed)	,941
Exact Sig. [2*(1-tailed Sig.)]	,943[b]

a. Grouping Variable: Group_OW_Ob

b. Not corrected for ties.

SE vs C

Test Statistics[a]

	Changes_HR
Mann-Whitney U	58,000
Wilcoxon W	178,000
Z	-1,590
Asymp. Sig. (2-tailed)	,112
Exact Sig. [2*(1-tailed Sig.)]	,126[b]

a. Grouping Variable: Group_OW_Ob

b. Not corrected for ties.

Ex vs Sm

Test Statistics[a]

	Changes_HR
Mann-Whitney U	72,000
Wilcoxon W	150,000
Z	-,886
Asymp. Sig. (2-tailed)	,376
Exact Sig. [2*(1-tailed Sig.)]	,399[b]

a. Grouping Variable: Group_OW_Ob

b. Not corrected for ties.

Ex vs C

Test Statistics[a]

	Changes_HR
Mann-Whitney U	90,000
Wilcoxon W	168,000
Z	,000
Asymp. Sig. (2-tailed)	1,000
Exact Sig. [2*(1-tailed Sig.)]	1,000[b]

a. Grouping Variable: Group_OW_Ob

b. Not corrected for ties.

Sm vs C

Test Statistics[a]

	Changes_HR
Mann-Whitney U	48,500
Wilcoxon W	126,500
Z	-1,383
Asymp. Sig. (2-tailed)	,167
Exact Sig. [2*(1-tailed Sig.)]	,178[b]

a. Grouping Variable: Group_OW_Ob

b. Not corrected for ties.

Figure 3C

SE vs Ex

Test Statistics[a]

	Changes_HR
Mann-Whitney U	71,500
Wilcoxon W	162,500
Z	-,672
Asymp. Sig. (2-tailed)	,502
Exact Sig. [2*(1-tailed Sig.)]	,511[b]

a. Grouping Variable: Group_OW_Ob

b. Not corrected for ties.

SE vs Sm

Test Statistics[a]

	Changes_HR
Mann-Whitney U	65,000
Wilcoxon W	143,000
Z	-,715
Asymp. Sig. (2-tailed)	,474
Exact Sig. [2*(1-tailed Sig.)]	,503[b]

a. Grouping Variable: Group_OW_Ob

b. Not corrected for ties.

SE vs C

Test Statistics[a]

	Changes_HR
Mann-Whitney U	33,000
Wilcoxon W	124,000
Z	-2,521
Asymp. Sig. (2-tailed)	,012
Exact Sig. [2*(1-tailed Sig.)]	,014[b]

a. Grouping Variable: Group_OW_Ob

b. Not corrected for ties.

Ex vs Sm

Test Statistics[a]

	Changes_HR
Mann-Whitney U	51,000
Wilcoxon W	129,000
Z	-1,475
Asymp. Sig. (2-tailed)	,140
Exact Sig. [2*(1-tailed Sig.)]	,152[b]

a. Grouping Variable: Group_OW_Ob

b. Not corrected for ties.

Ex vs C

Test Statistics[a]

	Changes_HR
Mann-Whitney U	47,000
Wilcoxon W	138,000
Z	-1,728
Asymp. Sig. (2-tailed)	,084
Exact Sig. [2*(1-tailed Sig.)]	,098[b]

a. Grouping Variable: Group_OW_Ob

b. Not corrected for ties.

Sm vs C

Test Statistics[a]

	Changes_HR
Mann-Whitney U	20,000
Wilcoxon W	98,000
Z	-3,046
Asymp. Sig. (2-tailed)	,002
Exact Sig. [2*(1-tailed Sig.)]	,002[b]

a. Grouping Variable: Group_OW_Ob

b. Not corrected for ties.

Figure 3D

SE vs Ex

Test Statistics[a]

	Changes_OBLA
Mann-Whitney U	261,500
Wilcoxon W	667,500
Z	-1,970
Asymp. Sig. (2-tailed)	,049

a. Grouping Variable: Group_TOTAL

Ex vs Sm

Test Statistics[a]

	Changes_OBLA
Mann-Whitney U	192,500
Wilcoxon W	598,500
Z	-2,461
Asymp. Sig. (2-tailed)	,014

a. Grouping Variable: Group_TOTAL

SE vs Sm

Test Statistics[a]

	Changes_OBLA
Mann-Whitney U	289,500
Wilcoxon W	667,500
Z	-,411
Asymp. Sig. (2-tailed)	,681

a. Grouping Variable: Group_TOTAL

Ex vs C

Test Statistics[a]

	Changes_OBLA
Mann-Whitney U	283,500
Wilcoxon W	559,500
Z	-,733
Asymp. Sig. (2-tailed)	,464

a. Grouping Variable: Group_TOTAL

SE vs C

Test Statistics[a]

	Changes_OBLA
Mann-Whitney U	177,000
Wilcoxon W	453,000
Z	-2,610
Asymp. Sig. (2-tailed)	,009

a. Grouping Variable: Group_TOTAL

Sm vs C

Test Statistics[a]

	Changes_OBLA
Mann-Whitney U	121,000
Wilcoxon W	397,000
Z	-3,165
Asymp. Sig. (2-tailed)	,002

a. Grouping Variable: Group_TOTAL

Figure 3E

SE vs Ex

Test Statistics[a]

	Changes_OBLA
Mann-Whitney U	72,000
Wilcoxon W	192,000
Z	-1,446
Asymp. Sig. (2-tailed)	,148
Exact Sig. [2*(1-tailed Sig.)]	,158[b]

a. Grouping Variable: Group_OW_Ob

b. Not corrected for ties.

Ex vs Sm

Test Statistics[a]

	Changes_OBLA
Mann-Whitney U	65,000
Wilcoxon W	185,000
Z	-1,225
Asymp. Sig. (2-tailed)	,221
Exact Sig. [2*(1-tailed Sig.)]	,236[b]

a. Grouping Variable: Group_OW_Ob

b. Not corrected for ties.

SE vs Sm

Test Statistics[a]

	Changes_OBLA
Mann-Whitney U	75,000
Wilcoxon W	153,000
Z	-,465
Asymp. Sig. (2-tailed)	,642
Exact Sig. [2*(1-tailed Sig.)]	,667[b]

a. Grouping Variable: Group_OW_Ob

b. Not corrected for ties.

Ex vs C

Test Statistics[a]

	Changes_OBLA
Mann-Whitney U	76,000
Wilcoxon W	154,000
Z	-,685
Asymp. Sig. (2-tailed)	,493
Exact Sig. [2*(1-tailed Sig.)]	,516[b]

a. Grouping Variable: Group_OW_Ob

b. Not corrected for ties.

SE vs C

Test Statistics[a]

	Changes_OBLA
Mann-Whitney U	43,000
Wilcoxon W	121,000
Z	-2,118
Asymp. Sig. (2-tailed)	,034
Exact Sig. [2*(1-tailed Sig.)]	,036[b]

a. Grouping Variable: Group_OW_Ob

b. Not corrected for ties.

Sm vs C

Test Statistics[a]

	Changes_OBLA
Mann-Whitney U	38,000
Wilcoxon W	116,000
Z	-1,972
Asymp. Sig. (2-tailed)	,049
Exact Sig. [2*(1-tailed Sig.)]	,052[b]

a. Grouping Variable: Group_OW_Ob

b. Not corrected for ties.

Figure 3F

SE vs Ex

Test Statistics[a]

	Changes_OBLA
Mann-Whitney U	56,500
Wilcoxon W	147,500
Z	-1,450
Asymp. Sig. (2-tailed)	,147
Exact Sig. [2*(1-tailed Sig.)]	,153[b]

a. Grouping Variable: Group_OW_Ob

b. Not corrected for ties.

Ex vs Sm

Test Statistics[a]

	Changes_OBLA
Mann-Whitney U	34,000
Wilcoxon W	125,000
Z	-2,181
Asymp. Sig. (2-tailed)	,029
Exact Sig. [2*(1-tailed Sig.)]	,030[b]

a. Grouping Variable: Group_OW_Ob

b. Not corrected for ties.

SE vs Sm

Test Statistics[a]

	Changes_OBLA
Mann-Whitney U	54,500
Wilcoxon W	145,500
Z	-,995
Asymp. Sig. (2-tailed)	,320
Exact Sig. [2*(1-tailed Sig.)]	,331[b]

a. Grouping Variable: Group_OW_Ob

b. Not corrected for ties.

Ex vs C

Test Statistics[a]

	Changes_OBLA
Mann-Whitney U	62,500
Wilcoxon W	128,500
Z	-,528
Asymp. Sig. (2-tailed)	,597
Exact Sig. [2*(1-tailed Sig.)]	,608[b]

a. Grouping Variable: Group_OW_Ob

b. Not corrected for ties.

SE vs C

Test Statistics[a]

	Changes_OBLA
Mann-Whitney U	41,500
Wilcoxon W	107,500
Z	-1,760
Asymp. Sig. (2-tailed)	,078
Exact Sig. [2*(1-tailed Sig.)]	,082[b]

a. Grouping Variable: Group_OW_Ob

b. Not corrected for ties.

Sm vs C

Test Statistics[a]

	Changes_OBLA
Mann-Whitney U	24,500
Wilcoxon W	90,500
Z	-2,384
Asymp. Sig. (2-tailed)	,017
Exact Sig. [2*(1-tailed Sig.)]	,016[b]

a. Grouping Variable: Group_OW_Ob

b. Not corrected for ties.

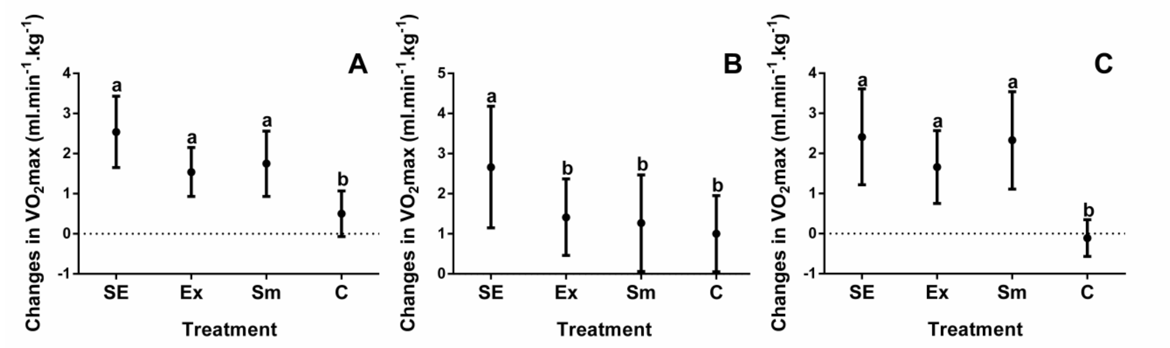

Figure 4. Changes in maximal oxygen uptake by treatments. VO_2max: Maximal oxygen uptake; SE: *Spirulina* and exercise; Ex: Exercise and placebo; Sm: *Spirulina* without exercise; and C: Control (Placebo treatment). (**A**) Total VO_2max changes of subjects; (**B**) VO_2max changes in overweight subjects; and (**C**) VO_2max changes in subjects with obesity. Data are shown as the mean, with 95% confidence intervals. Different letters indicate the statistical differences between groups (p < 0.05).

Figure 4A

SE vs Ex

Test Statistics[a]

	Changes_VO2 max
Mann-Whitney U	213,000
Wilcoxon W	513,000
Z	-1,580
Asymp. Sig. (2-tailed)	,114

a. Grouping Variable: Group_TOTAL

SE vs Sm

Test Statistics[a]

	Changes_VO2 max
Mann-Whitney U	194,000
Wilcoxon W	404,000
Z	-1,098
Asymp. Sig. (2-tailed)	,272

a. Grouping Variable: Group_TOTAL

SE vs C

Test Statistics[a]

	Changes_VO2 max
Mann-Whitney U	101,000
Wilcoxon W	311,000
Z	-3,340
Asymp. Sig. (2-tailed)	,001

a. Grouping Variable: Group_TOTAL

Ex vs Sm

Test Statistics[a]

	Changes_VO2 max
Mann-Whitney U	219,000
Wilcoxon W	519,000
Z	-,506
Asymp. Sig. (2-tailed)	,613

a. Grouping Variable: Group_TOTAL

Ex vs C

Test Statistics[a]

	Changes_VO2 max
Mann-Whitney U	138,000
Wilcoxon W	348,000
Z	-2,481
Asymp. Sig. (2-tailed)	,013

a. Grouping Variable: Group_TOTAL

Sm vs C

Test Statistics[a]

	Changes_VO2 max
Mann-Whitney U	114,000
Wilcoxon W	324,000
Z	-2,373
Asymp. Sig. (2-tailed)	,018
Exact Sig. [2*(1-tailed Sig.)]	,020[b]

a. Grouping Variable: Group_TOTAL

b. Not corrected for ties.

Figure 4B

SE vs Ex

Test Statistics[a]

	Changes_VO2 max
Mann-Whitney U	50,000
Wilcoxon W	128,000
Z	-1,299
Asymp. Sig. (2-tailed)	,194
Exact Sig. [2*(1-tailed Sig.)]	,219[b]

a. Grouping Variable: Group_OW_Ob

b. Not corrected for ties.

Ex vs Sm

Test Statistics[a]

	Changes_VO2 max
Mann-Whitney U	60,000
Wilcoxon W	126,000
Z	-,378
Asymp. Sig. (2-tailed)	,705
Exact Sig. [2*(1-tailed Sig.)]	,740[b]

a. Grouping Variable: Group_OW_Ob

b. Not corrected for ties.

SE vs Sm

Test Statistics[a]

	Changes_VO2 max
Mann-Whitney U	43,000
Wilcoxon W	109,000
Z	-1,434
Asymp. Sig. (2-tailed)	,152
Exact Sig. [2*(1-tailed Sig.)]	,169[b]

a. Grouping Variable: Group_OW_Ob

b. Not corrected for ties.

Ex vs C

Test Statistics[a]

	Changes_VO2 max
Mann-Whitney U	58,000
Wilcoxon W	124,000
Z	-,505
Asymp. Sig. (2-tailed)	,613
Exact Sig. [2*(1-tailed Sig.)]	,651[b]

a. Grouping Variable: Group_OW_Ob

b. Not corrected for ties.

SE vs C

Test Statistics[a]

	Changes_VO2 max
Mann-Whitney U	41,000
Wilcoxon W	107,000
Z	-1,560
Asymp. Sig. (2-tailed)	,119
Exact Sig. [2*(1-tailed Sig.)]	,134[b]

a. Grouping Variable: Group_OW_Ob

b. Not corrected for ties.

Sm vs C

Test Statistics[a]

	Changes_VO2 max
Mann-Whitney U	55,500
Wilcoxon W	121,500
Z	-,334
Asymp. Sig. (2-tailed)	,738
Exact Sig. [2*(1-tailed Sig.)]	,748[b]

a. Grouping Variable: Group_OW_Ob

b. Not corrected for ties.

Figure 4C

SE vs Ex

Test Statistics[a]

	Changes_VO2 max
Mann-Whitney U	55,500
Wilcoxon W	133,500
Z	-,977
Asymp. Sig. (2-tailed)	,329
Exact Sig. [2*(1-tailed Sig.)]	,347[b]

a. Grouping Variable: Group_OW_Ob

b. Not corrected for ties.

Ex vs Sm

Test Statistics[a]

	Changes_VO2 max
Mann-Whitney U	39,500
Wilcoxon W	117,500
Z	-1,075
Asymp. Sig. (2-tailed)	,282
Exact Sig. [2*(1-tailed Sig.)]	,310[b]

a. Grouping Variable: Group_OW_Ob

b. Not corrected for ties.

SE vs Sm

Test Statistics[a]

	Changes_VO2 max
Mann-Whitney U	53,500
Wilcoxon W	131,500
Z	-,036
Asymp. Sig. (2-tailed)	,971
Exact Sig. [2*(1-tailed Sig.)]	,972[b]

a. Grouping Variable: Group_OW_Ob

b. Not corrected for ties.

Ex vs C

Test Statistics[a]

	Changes_VO2 max
Mann-Whitney U	10,500
Wilcoxon W	55,500
Z	-3,222
Asymp. Sig. (2-tailed)	,001
Exact Sig. [2*(1-tailed Sig.)]	,001[b]

a. Grouping Variable: Group_OW_Ob

b. Not corrected for ties.

SE vs C

Test Statistics[a]

	Changes_VO2 max
Mann-Whitney U	9,500
Wilcoxon W	54,500
Z	-3,270
Asymp. Sig. (2-tailed)	,001
Exact Sig. [2*(1-tailed Sig.)]	,001[b]

a. Grouping Variable: Group_OW_Ob

b. Not corrected for ties.

Sm vs C

Test Statistics[a]

	Changes_VO2 max
Mann-Whitney U	1,500
Wilcoxon W	46,500
Z	-3,550
Asymp. Sig. (2-tailed)	,000
Exact Sig. [2*(1-tailed Sig.)]	,000[b]

a. Grouping Variable: Group_OW_Ob

b. Not corrected for ties.
